# Keystone Veterinary Medical Association

**Published:** 1897-11

**Authors:** W. L. Rhoads

**Affiliations:** Secretary


Secretary.

### KEYSTONE VETERINARY MEDICAL ASSOCIATION.

THE September meeting was called to order by Dr. James B. Rayner, Tuesday evening, the 13th, at 8.30 o'clock, in Room No. 1, third floor, northwest corner Broad and Filbert Streets, with the following members of the profession present: W. H. Hoskins, Charles Lintz, James T. McAnulty, James B. Rayner, W. L. Rhoads, Thomas B. Rayner, William H. Ridge, C. J. Marshall; also students from the Veterinary Department of the University of Pennsylvania. Upon the recommendation of the Board of Trustees, Dr. Ridge was unanimously reinstated as an active member.

Dr. James T. McAnulty now gave a very comprehensive talk upon what the various schools of farriery intend doing throughout the coming winter sessions, outlining, first, the course of instruction to be given, then the form of examination, and a number of questions as an example of what would be asked, the code of ethics which all must sign upon their graduation, and then he portrayed the undoubted advantages that must necessarily accrue to the workers of the craft.

He asked for the assistance and coöperation of the veterinary profession in the good work of the master horseshoers. The Association extended to the master horseshoers, through Dr. McAnulty, its fraternal sympathies, encouragement, and support.

We then heard from those who had the good fortune to be at the national meeting at Nashville. They all felt that the ride was remarkable for dirt, yet they to a man agreed that it was the only low-down, mean part of the trip, as they were met at every turn with the renowned, open-armed hospitality often talked of, but seen in its most generous and perfect form in our own Southern States. The honor of being elected Eastern Vice-President having fallen upon one of our members, he was now called on to tell of his experiences and emotions. In conclusion, he promised, while enacting his official duties, to be at all times sure of his premises before proceeding, thus opening no possible chance for grievance from any brother.

It was now moved and seconded that Dr. Ridge prepare a paper for our October meeting.

Dr. James B. Rayner admonished the members to be more prompt in attendance and time of arrival, that the meetings might always be called to order at 8 o'clock sharp, thus giving time to carry out the programme before any had to leave for home.

Dr. Ridge then reported several cases of pyæmia in cattle, caused by scratches from cut briers and stagnant water through which they passed.

Meeting then adjourned to meet October 12th. This will be the annual meeting. Officers will be elected and some Association news of interest to members will be made known.

THE October meeting of the Association was called to order by Dr. Thomas B. Rayner, who took the chair, upon motion of Dr. Hoskins, in the absence of President James B. Rayner.

The following members of the profession were present: Drs. F. S. Allen, Charles M. Cullen, Charles T. Goentner, Walter L. Hart, W. Horace Hoskins, W. S. Kooker, Charles Lintz, Leonard Pearson, W. L. Rhoads, Thomas B. Rayner, H. A. Christman, William H. Ridge, C. J. Marshall, John W. Adams, J. C. Michener, Robert Gladfelter, J. C. Foelker, S. J. J. Harger, J. Beatty, and E. M. Ranck; also Messrs. P. K. Jones, J. E. Spindler, A. E. Cunningham, S. L. Blount, L. D. Horner, C. Megary, J. J. Repp, H. Hoopes, and others whose names we did not get.

Dr. J. C. Michener, of Colmar, read a paper on the "Value of Exercise." He gave one of the most forceful papers, written upon independent, free lines of thought and experience, regardless of text-books and like authority, that it has been the favor of the Association to hear for some time. He said exercise out in the open air and sunlight was a most essential requisite to health. Thus we procure keenest appetites and enjoyment, accelerate and deepen circulation and respiration; tone and strength are given to every organ; thus we promote secretions and excretions. When neglected, stagnation and impurity begin, and, if habitual, disease is invited and life shortened. He then told of the value of exercise in strangles, grease-heel, influenza, dumb staggers, and laminitis in its early stages, congestive pneumonia, azoturia, etc., and went into details to tell why and in just what way the exercise was helpful in each case, and how they were put upon their feet and started when found down. He dwelt especially upon the value of good sanitation, good ventilation, and plenty of light. He defended his views in the discussion, citing a number of cases where one would think movement impossible; he has proven it not only to be practical, but found it to be essential to a rapid recovery. He concluded by saying we must not expect an animal to have perfect health or be restored, when sick, if confined in a dark place without exercise. The discussion was interesting, bringing forth many good points and numerous opinions.

Dr. William R. Ridge now read his paper on "Acute Indigestion in the Cow." This paper also showed much thought and thorough work on the part of the author. The discussion on this paper was taken part in by each of those in attendance, and would have taken up considerable space, but was closed, that our annual election might be held.

The results of said election were as follows: President, Leonard Pearson; Vice-President, Hile P. Eves; Treasurer, Francis Bridge; Secretary, W. L. Rhoads; Censors, William H. Ridge, W. Horace Hoskins, W. S. Kooker, T. B. Rayner, and Charles Lintz.

President-elect Pearson was now called to the chair, the duties of which he accepted in a short, expressive, and appropriate speech.

Dr. Hoskins spoke now of the present meat-inspection of Philadelphia and possible means of improvement. He then made a motion that the President appoint a committee of three to confer with the Woman's Health Protective Association and other kindred organizations in an endeavor to bring about a better system of meat-inspection.. The President appointed as committee Drs. W. H. Hoskins, John W. Adams, and F. S. Allen.

Dr. S. J. J. Harger now reported several cases of open joint, with discharge of synovia in the cases where the animal at the same time was affected with strangles; the recovery was most rapid and complete. He wanted to know if there was a specific action upon the joint due to the streptococci causing the latter trouble. The discussion was necessarily short, as the meeting now adjourned, postponing the report of delegates from Franklin until November 9th. DR. W. L. RHOADS,

Secretary.

